# Risk Factors for the Development of Eating Disorders in Adolescents with Early-Onset Inflammatory Bowel Diseases

**DOI:** 10.3390/nu16162675

**Published:** 2024-08-13

**Authors:** Anna Riva, Gabriele Arienti, Giovanna Zuin, Laura Spini, Margherita Calia, Andrea Biondi, Renata Nacinovich, Andrea E. Cavanna

**Affiliations:** 1 Department of Child Neuropsychiatry, Fondazione IRCCS San Gerardo dei Tintori, 20900 Monza, Italy; anna.riva@irccs-sangerardo.it (A.R.); gabriele.arienti@irccs-sangerardo.it (G.A.); l.spini4@campus.unimib.it (L.S.); 2 School of Medicine and Surgery, University of Milano-Bicocca, 20900 Monza, Italy; andrea.biondi@unimib.it (A.B.); a.e.cavanna@bham.ac.uk (A.E.C.); 3 School of Medicine and Surgery, University of Brescia, 25121 Brescia, Italy; 4 Department of Pediatrics, Fondazione IRCCS San Gerardo dei Tintori, 20900 Monza, Italy; giovanna.zuin@irccs-sangerardo.it (G.Z.); margherita.calia@irccs-sangerardo.it (M.C.); 5 Department of Neuropsychiatry, BSMHFT, University of Birmingham, Birmingham B15 2TT, UK; 6 School of Life and Health Sciences, Aston Brain Centre, Aston University, Birmingham B15 2TT, UK; 7 Sobell Department of Motor Neuroscience and Movement Disorders, Institute of Neurology, University College London, London WC1E 6BT, UK

**Keywords:** inflammatory bowel diseases, eating disorders, adolescents, alexithymia, psychological symptoms

## Abstract

Individuals with inflammatory bowel diseases (IBDs) have an increased risk of developing psychiatric comorbidities, including eating disorders (EDs). We aimed to investigate the potential association between key disease characteristics, including psychological features, and the development of EDs in a clinical sample of adolescents with IBDs. We enrolled 52 adolescents with IBDs, 83% of whom were in clinical remission, and systematically collected additional information on disease duration, the total number of relapses, the use of steroids, and the number of hospital admissions. All participants completed a validated psychometric battery assessing psychological symptoms (Symptom Checklist-90–Revised, SCL-90-R), alexithymia (Toronto Alexithymia Scale-20, TAS-20), and ED symptomatology (Eating Disorders Inventory-3rd edition, EDI-3). About one in ten patients (9.6%) reported Eating Disorder Risk scores higher than the cut-off on the EDI-3 subscale, specifically addressing the risk of developing EDs. According to the EDI-3 scores, the risk of developing EDs directly correlated with the number of total relapses of IBDs (*p* < 0.05). The TAS-total scores also correlated with the number of total relapses (*p* < 0.01), as well as with the number of steroid cycles (*p* < 0.05), the number of hospital admissions (*p* < 0.05), and overall disease duration (*p* < 0.05). Our findings suggest that disease relapses increase the risk of developing both EDs and alexithymia in adolescents with IBDs. The recurrence of disease relapses should be identified and screened early on to prevent the onset of psychiatric disorders, including EDs. Research should be conducted on larger samples with different IBD phenotypes to further investigate the characteristics of patients with IBDs at risk of developing EDs.

## 1. Introduction

Inflammatory bowel diseases (IBDs) are chronic inflammatory disorders of the gastrointestinal tract with a steadily increasing incidence and prevalence worldwide, mainly in the pediatric population [1]. A recent systematic review reported prevalence rates of pediatric-onset IBDs ranging from 5.0 to 52.2 per 100,000 in Asia, from 31.0 to 75.0 in Europe, from 28.3 to 63.6 in Canada, and from 21.7 to 46.0 in Oceania [1]. The different forms of IBDs include Crohn's disease (CD), ulcerative colitis (UC), and IBD unclassified (IBD-U) [2]. The current conceptualization of IBDs as a spectrum of pathological conditions, as well as recent advances in monitoring and treatment strategies, has significantly improved the prognosis of these disorders [2]. However, the characteristic relapsing–remitting course of IBDs and a longer disease duration have been associated with the onset of psychological symptoms, especially during exacerbations [3,4].

Psychiatric comorbidities such as anxiety disorders, major depression, and suicide attempts have been reported in individuals with IBDs, including children and adolescents [5]. Psychological distress manifesting with a broader range of psychiatric symptoms has recently been documented in adolescents with IBDs compared to their peers [6]. Moreover, research on alexithymia (the inability to be aware of, explicitly identify, and describe one's feelings) has revealed correlations with poorer mental health and clinical outcomes in adults with IBDs [4]. However, these findings have not been confirmed in adolescent samples.

Individuals with IBDs have an increased risk of developing eating disorders (EDs), sharing many of the established risk factors for EDs: younger age, anxiety and mood disorders, body image dissatisfaction, chronic health problems, and the use of restrictive diets [7,8]. In clinical practice, it has been observed that patients with IBDs commonly associate their diet with symptoms and inquire about dietary modifications to manage their IBDs [9]. In this regard, specific dietary regimes (for example, Crohn's disease exclusion diet, diet with specific carbohydrates, Mediterranean diet, low FODMAP diet, and anti-inflammatory diet) are sometimes used as therapeutic options for individuals with IBDs in different disease stages, with varying degrees of efficacy [9]. There is evidence suggesting that IBDs and EDs share common pathogenetic mechanisms, including autoimmunity processes, microbiome alterations, and gastrointestinal system–brain axis dysregulation, prompting further research on the relationship between the two conditions [10].

Little is known about the possible associations between the clinical features of IBDs (including psychological symptoms) and the development of EDs, especially in youths [11]. Active disease and a shorter disease duration seem to predict anxiety and depressive symptoms [11]; however, to date, few studies have been conducted to evaluate disease-related and psychological risk factors for EDs.

Therefore, we aimed to investigate the potential association between key disease characteristics, including psychological features, and the development of EDs in a clinical sample of adolescents with IBDs.

## 2. Materials and Methods

### 2.1. Clinical Sample

We assessed 75 adolescents aged 12 to 17 with a diagnosis of IBD confirmed by a combination of endoscopic, radiological, biochemical, and histological investigation, according to the ESPGHAN Revised Porto Criteria [12]. All clinical assessments were carried out at the tertiary Pediatric Gastroenterology Unit, Department of Pediatrics, Fondazione IRCCS San Gerardo dei Tintori (Monza, Italy), between October 2022 and February 2023. The exclusion criteria were previous psychological or psychiatric disorders, physical comorbidities, intellectual disabilities, substance/alcohol abuse, and insufficient proficiency in the Italian language. A total of 23 patients were excluded from our study: 8 subjects with psychiatric comorbidities, 5 with insufficient comprehension of the Italian language, and 10 who refused to participate. As a result, from the initial pool, 52 patients (44% females) were eventually enrolled. Our clinical sample of IBD subjects consisted of 33 patients with UC and 19 patients with CD.

All adolescents and their parents were provided with information about the purpose of this study and written informed consent was obtained from the participants’ parents. This study received ethical approval from the Brianza Ethics Committee (Protocol code: 311 29/06/2023) and was conducted in accordance with the principles of the Declaration of Helsinki (1964) and its subsequent amendments.

### 2.2. Measures

An ad hoc questionnaire was designed to gather demographic details, including age, gender, and body mass index (BMI) at diagnosis and at the time of test evaluation. The anthropometric parameters, including height, weight, and BMI, were obtained during a physical examination both at diagnosis and at the time of test evaluation. Weight, height, and BMI, defined as weight divided by height squared (kg/m^2^), were calculated as absolute values as well as *z*-scores. The BMI *z*-score was calculated according to the Italian reference growth charts specific for age and gender [13]. Family socio-economic status (SES) was assessed using the Hollingshead four-factor index [14]. We systematically collected additional information on disease duration, the number of total relapses (both during the entire course of disease and in the last 6 months), the use of steroids (both during the entire course of disease and in the last 6 months), and the number of hospital admissions. Clinical remission status at the time of evaluation was assessed using either the Pediatric Crohn's Disease Activity Index (PCDAI) [15,16] or the Pediatric Ulcerative Colitis Activity Index (PUCAI) [17,18].

In addition to providing sociodemographic and clinical data, all participants completed a psychometric battery comprising the Italian versions of validated measures of psychological symptoms [19], alexithymia [20], and eating symptomatology [21] within three months of receiving an IBD diagnosis.

The Symptom Checklist-90–Revised (SCL-90-R) is a self-report questionnaire designed to assess psychological problems and psychopathological symptoms in individuals aged 12 years and older. The SCL-90-R consists of 90 items rated on a 5-point Likert scale that assess nine symptom dimensions: somatization (SOM), obsessive–compulsive (O-C), interpersonal sensitivity (I-S), depression (DEP), anxiety (ANX), hostility (HOS), phobic anxiety (PHOB), paranoid ideation (PAR), and psychoticism (PSY). The SCL-90-R includes a Global Severity Index (GSI), which quantifies the risk of developing psychiatric disorders; scores between 55 and 65 are considered borderline, whereas scores higher than 65 suggest pathology. The Italian version of the SCL-90-R showed good internal coherence for all subscales (α values between 0.70 and 0.96) [19].

The Toronto Alexithymia Scale-20 (TAS-20) is a 5-point Likert-type self-report questionnaire consisting of 20 items that assess alexithymia across three factors: Difficulty Identifying Feelings (DIF), Difficulty Describing Feelings (DDF), and Lack of Focus on Internal Emotional Experiences (EOT). A total score of at least 61 indicates the presence of alexithymia tracts. The Italian version of the TAS-20 is characterized by good internal consistency (Cronbach’s α 0.75 and 0.82 in normal and clinical groups, respectively) and high test–retest reliability over 2 weeks (r = 0.86). A confirmatory factor analysis revealed the same factor structure as the original English version and adequate internal consistency of the subscales, with α coefficients equal to or greater than 0.70 [20].

The Eating Disorders Inventory-3rd edition (EDI-3) is a self-report instrument measuring psychological traits associated with EDs. This instrument comprises 91 items organized into three ED-specific scales and nine general psychological scales relevant to (but not specific to) EDs. Furthermore, the EDI-3 includes six composite scales. The Eating Disorder Risk (EDRC) scale specifically addresses the risk of developing EDs. The other five scales explore general integrative psychological constructs: Ineffectiveness (IC), Interpersonal Problems (IPCs), Affective Problems (APs), Overcontrol (OC), and General Psychological Maladjustment Composite (GPMC). Scores higher than 70 are considered above the cut-off for clinical risk. The reliability coefficients of the scales range from 0.80 to 0.90, and test–retest reliability coefficients for the various composite scales are between 0.93 and 0.98 [21].

### 2.3. Data Analysis

Categorical variables are expressed as absolute values and percentages, whereas continuous variables are expressed as medians and skewness as well as means and standard deviations, since the data were not normally distributed. Spearman’s correlations have been calculated to study the possible associations between disease features and psychometric scores. Variables with significant associations have been entered into linear regression analyses. All statistical analyses were performed using the IBM SPSS 28 statistical software package, with the significance level set at *p* values < 0.05.

## 3. Results

The sociodemographic and disease characteristics of the clinical sample of adolescents with IBDs are summarized in Table 1. Most patients (82.7%) were in clinical remission (defined as either a PCDAI score ≤ 10 or a PUCAI score < 10). Approximately one in ten patients (9.6%) reported EDI-EDRC scores higher than the cut-off for a clinical risk of developing EDs.

Spearman’s correlation coefficients between disease features and psychometric scores are shown in Table 2. The risk of developing EDs as assessed by EDI-EDRC scores was directly correlated with the total number of IBD relapses (*p* < 0.05).

Alexithymia, as assessed by TAS-20 scores, was directly correlated with the total number of IBD relapses (*p* < 0.01), the total number of steroid cycles (*p* < 0.05), the number of hospital admissions (*p* < 0.05), and disease duration (*p* < 0.05).

Finally, there was a significant correlation between paranoid ideation (as assessed by SCL-90-R PAR scores) and the number of hospital admissions (*p* < 0.05).

The regression model with EDI-EDRC score as the dependent variable and the total number of relapses as the predictor reached statistical significance, explaining 12.4% of the variability in the risk of developing EDs (F = 6.660, *p* = 0.013). Specifically, the total number of relapses (B = 5.486, SE = 2.125) was identified as a significant predictor of the risk of developing EDs.

The regression model with TAS-20 total score as the dependent variable and the total number of relapses as the predictor also reached statistical significance, explaining 17.4% of the variability in the risk of developing alexithymia (F = 10.529, *p* = 0.002). Specifically, the total number of relapses (B = 2.608, SE = 0.804) was identified as a significant predictor of the risk of developing alexithymia. The number of steroid cycles and the number of hospital admissions were both excluded from the regression model because of their strong correlation with the number of disease relapses (*p* < 0.001).

## 4. Discussion

To the best of our knowledge, we presented original data from the first observational study of risk factors for the development of EDs in a clinical sample of adolescents with IBDs. In our study, the proportion of adolescents with IBDs at risk of developing EDs was 9.6%, a figure that is in line with previous studies on adolescents (10%) [22] and adults (13%) [8]. We found evidence for a direct association between the number of IBD relapses and the risk of developing EDs. A possible explanation is that gastrointestinal symptoms are especially common during exacerbation phases and can influence and/or precipitate EDs. For example, a prospective questionnaire-based study of 400 adults with IBDs in the United Kingdom showed that 57% of patients felt that diet could influence their condition by triggering relapses, with two-thirds of them depriving themselves of their favorite food to achieve symptom control [23].

In our study, the number of IBD relapses, resulting in pharmacotherapy with steroids and hospital admissions, was found to contribute to the development of alexithymia symptoms. Previous studies reported an increased prevalence of alexithymia in patients with IBDs [24]. Moreover, an alexithymic trait has been linked to increased psychiatric comorbidity and greater impairment of health-related quality of life. Extended gastrointestinal involvement, prolonged duration of undiagnosed pathology, and overall IBD severity have been associated with the development of alexithymia in adult patients [25].

To the best of our knowledge, the association between disease features and alexithymia in pediatric IBD samples has not been previously investigated. Our study provides initial evidence that the number of IBD relapses, an indicator of disease severity, can predict the development of alexithymia in this age group. Of note, in our study, other disease features were not associated with psychological symptoms, in agreement with the results of a previous clinical study on a pediatric sample affected by Crohn’s disease [26]. This finding is also supported by the observation that the treatment of depression in adolescents with IBDs is not associated with changes in disease severity [27]. A possible explanation is that patients with IBDs might present with higher levels of psychological distress, regardless of the disease course [28]. Despite significant improvement in the treatment of IBDs, these conditions are still characterized by an unpredictable course. Furthermore, the onset of IBDs in the pediatric age can lead to increased psychological distress because their clinical manifestations and implications often cause real and/or perceived stigmatization, which is prevalent in this age group [26].

## 5. Conclusions

The present study has limitations. Because of the relatively small sample size, it was not possible to conduct subgroup analyses on the different phenotypes of IBDs. Moreover, since we recruited our research sample from a single specialist clinic, referral bias might limit the generalizability of our findings. Despite these limitations, our findings suggest that disease relapses increase the risk of developing both EDs and alexithymia in adolescents with IBDs. In turn, alexithymia can be associated with an increased risk of presenting with subsequent psychiatric comorbidities. Therefore, disease relapses in adolescents with IBDs should be screened for and detected at an early stage in order to prevent the onset of psychiatric disorders, including EDs. Further research is needed to replicate our findings in larger samples, including across multiple clinical sites to enhance the representativeness of the findings. Subgroup analyses of specific phenotypes of IBDs would improve the clinical characterization of patients with IBDs at risk of developing EDs. Longitudinal studies are needed to better elucidate the temporal relationships between disease characteristics and the development of psychological features, including EDs. Finally, it is important that the research agenda incorporates clinical studies exploring the potential role of other relevant factors, such as family history, socio-economic status, and specific treatment modalities, in the risk of developing EDs.

## Figures and Tables

**Table 1:** Sociodemographic and disease features and psychometric scores in adolescents with IBDs (N = 52).

Sociodemographic and Disease Features	N (%)	Mean (SD)	Median (Skewness)
**Age**		15.4 (1.97)	16.0 (−0.467)
**Female gender**	23 (44.3)		
**Socio-economic status**		31.2 (11.72)	28.0 (0.746)
**Clinical remission at the time of test evaluation**	43 (82.7)		
**BMI at diagnosis**		17.8 (3.31)	17.4 (0.755)
**BMI *z*-score at diagnosis** **BMI at evaluation** **BMI *z*-score at evaluation**		−1.036 (1.26) 20.1 (3.06) −0.534 (1.06)	−0.920 (−0.469) 19.9 (0.785) −0.700 (0.324)
**N of relapses**		1.38 (1.89)	0.1 (1.625)
**N of relapses in the last 6 months**		0.25 (0.59)	0.0 (2.868)
**N of steroid cycles**		1.1 (1.46)	1.0 (1.920)
**N of hospital admissions**		1.0 (1.23)	1.0 (1.410)
**Psychometric scales**			
**SCL-90-R SOM**		48.8 (10.51)	47.0 (1.179)
**SCL-90-R O-C**		49.4 (10.58)	50.0 (0.798)
**SCL-90-R I-S**		48.9 (11.28)	46.5 (0.877)
**SCL-90-R DEP**		50.3 (10.33)	51.0 (0.605)
**SCL-90-R ANX**		48.8 (10.02)	46.0 (1.305)
**SCL-90-R HOS**		47.9 (10.70)	43.0 (0.706)
**SCL-90-R PHOB**		51.2 (10.83)	47.0 (1.341)
**SCL-90-R PAR**		48.2 (10.56)	48.0 (0.706)
**SCL-90-R PSY**		47.4 (8.64)	45.0 (1.705)
**SCL-90-R GSI**		48.8 (10.47)	48.0 (1.069)
**TAS-20**		50.4 (11.82)	52.5 (−0.170)
**EDI-EDRC**		32.5 (24.81)	25.0 (0.731)
**EDI-IC**		43.2 (26.68)	39.0 (0.144)
**EDI-IPC**		49.9 (25.51)	50.0 (−0.192)
**EDI-APC**		48.9 (27.01)	49.0 (0.101)
**EDI-OC**		45.2 (26.00)	42.0 (0.203)
**EDI-GPMC**		50.2 (21.94)	52.0 (−0.153)

Abbreviations: IBDs, inflammatory bowel diseases; BMI, body mass index; SCL-90-R, Symptom Checklist-90–Revised; SOM, somatization; O-C, obsessive–compulsive; I-S, interpersonal sensitivity; DEP, depression; ANX, anxiety; HOS, hostility; PHOB, phobic anxiety; PAR, paranoid ideation; PSY, psychoticism; GSI, Global Severity Index; TAS-20, Toronto Alexithymia Scale-20; EDI, Eating Disorder Inventory; EDRC, Eating Disorder Risk Composite; IC, Ineffectiveness Composite; IPC, Interpersonal Problem Composite; APC, Affective Problem Composite; OC, Overcontrol Composite; GPMC, General Psychological Maladjustment Composite.

**Table 2:** Spearman’s correlation coefficients between disease characteristics and psychometric scores in adolescents with IBDs (N = 52).

	BMI Z SCORE AT DIAGNOSIS	BMI Z SCORE AT TEST COMPLETION	N OF RELAPSES	N OF RELAPSES IN THE LAST 6 MONTHS	N OF STEROID CYCLES	N OF STEROID CYCLES IN THE LAST 6 MONTHS	N OF HOSPITAL ADMISSIONS	DISEASE DURATION (MONTHS)
SCL-90-R SOM	0.182	0.226	0.169	0.082	0.220	0.187	0.014	0.121
SCL-90-R O-C	0.087	0.002	0.106	0.137	0.099	0.043	−0.103	0.007
SCL-90-R I-S	0.209	0.195	0.080	0.060	0.047	0.043	−0.223	0.054
SCL-90-R DEP	0.187	0.083	0.123	0.192	0.120	0.094	−0.135	0.028
SCL-90-R ANX	0.202	0.078	0.081	0.097	0.091	0.068	−0.171	0.093
SCL-90-R HOS	0.022	0.105	−0.055	−0.080	0.117	−0.007	−0.081	0.073
SCL-90-R PHOB	0.045	0.038	0.024	0.035	−0.046	0.166	−0.191	0.010
SCL-90-R PAR	0.170	0.085	−0.079	0.021	−0.072	−0.137	−0.286 *	−0.056
SCL-90-R PSY	0.009	0.079	0.132	0.127	0.075	−0.012	−0.122	0.170
SCL-90-R GSI	0.162	0.160	0.056	0.104	0.089	0.064	−0.187	0.580
TAS-20	0.016	−0.026	0.406 **	0.017	0.348 *	−0.064	0.326 *	0.022 *
EDI-EDRC	0.297	0.182	0.345 *	−0.012	0.198	−0.133	0.191	0.272
EDI-IC	0.043	0.021	0.191	0.062	0.069	−0.100	−0.118	0.056
EDI-IPC	0.268	0.163	0.160	−0.145	0.213	−0.153	0.082	0.111
EDI-APC	−0.038	−0.048	0.248	−0.006	0.234	−0.094	0.164	0.174
EDI-OC	0.052	0.133	0.111	0.051	0.156	0.102	−0.264	−1.117
EDI-GPMC	0.016	0.030	0.202	−0.085	0.173	−0.123	−0.055	0.075

Abbreviations: IBDs, inflammatory bowel diseases; BMI, body mass index; SCL90-R, Symptom Checklist-90–Revised; SOM, somatization; O-C, obsessive–compulsive; I-S, interpersonal sensitivity; DEP, depression; ANX, anxiety; HOS, hostility; PHOB, phobic anxiety; PAR, paranoid ideation; PSY, psychoticism; GSI, Global Severity Index; TAS-20, Toronto Alexithymia Scale; EDI, Eating Disorder Inventory; EDRC, Eating Disorder Risk Composite; IC, Ineffectiveness Composite; IPC, Interpersonal Problem Composite; APC, Affective Problem Composite; OC, Overcontrol Composite; GPMC, General Psychological Maladjustment Composite. * *p* < 0.05 and ** *p* < 0.01.

## Data Availability

The data presented in this study are available on request from the corresponding author.
